# Diagnostic assessment of Fetal Alcohol Spectrum Disorder in adults

**DOI:** 10.1192/j.eurpsy.2026.11107

**Published:** 2026-07-31

**Authors:** C. M. Rehnquist, M. Abel, M. Reichl, T. Ohly, F. Kiefer, B. Lenz

**Affiliations:** 1 Department of Addictive Behavior and Addiction Medicine, Central Institute for Mental Health, Mannheim; 2 German Center for Mental Health, Mannheim-Heidelberg-Ulm, Germany

## Abstract

**Introduction:**

Fetal Alcohol Spectrum Disorder (FASD) is frequently diagnosed in childhood but is often missed or misdiagnosed in adulthood due to age-related changes. Adults with FASD face a wide range of challenges, including psychiatric and somatic comorbidities, difficulties in education, employment, and social participation. Specialized diagnostic services for adults with suspected FASD remain scarce in Germany.

**Objectives:**

To present initial results from the newly established FASD specialist outpatient service for adults at the Central Institute of Mental Health in Mannheim, Germany.

**Methods:**

The FASD service for adults was launched in 2024 and diagnoses adults with suspected FASD. An interdisciplinary team of physicians and psychologists perform standardized diagnostic assessments. The poster will describe the diagnostic workflow based on the German consent for adults published by Schecke et al. (Schecke et al. SUCHT 2024; 70:275-284) and present early cohort data. We will provide data on patients who received services from us in 2024 and 2025.

**Results:**

Data is currently being analyzed. Results to be presented at the congress will include the diagnostic distribution (Fetal Alcohol Syndrome (FAS), partial FAS (pFAS), Alcohol-Related Neurodevelopmental Disorder (ARND), frequencies of facial and growth abnormalities, birth-related data, and summary statistics from cognitive and neuropsychological testing.

**Conclusions:**

The FASD service for adults addresses an important gap in care by providing targeted diagnostic assessment and counselling. Early experience highlights substantial need and supports the relevance of specialized services to improve identification and care for adults with FASD.

**Disclosure of Interest:**

None Declared

